# Comparative efficacy and studies of mode of action of minerals from diatoms against three species of filth flies

**DOI:** 10.1111/mve.70052

**Published:** 2026-01-30

**Authors:** Grayson L. Cave, Kaiying Chen, Steven S. Denning, David W. Watson, R. Michael Roe

**Affiliations:** ^1^ Department of Entomology and Plant Pathology North Carolina State University Raleigh North Carolina USA

**Keywords:** DE, diatomaceous earth, expanded perlite, filth flies, mechanical insecticides, non‐chemical, pest control

## Abstract

Filth flies pose a health risk because of the microbes they carry outside and inside of their bodies to humans and animals. Mostly synthetic chemical insecticides are used for fly control. Alternative approaches with a different mode of action are needed because of increasing fly resistance to pesticides. We used a modified World Health Organization cone test to determine the efficacy of the minerals produced by diatoms (diatomaceous earth) as a mechanical insecticide against adults of the house fly, *Musca domestica*, the secondary screwworm, *Cochliomyia macellaria,* and the grey flesh fly, *Sarcophaga bullata*, under low and high humidity. The use of mechanical insecticides as an alternative to kill filth flies has received minimal consideration. However, recent research showed that expanded perlite, a new mechanical insecticide made from volcanic rock, was highly efficacious against mosquitoes and flies. Mortality for diatomaceous earth in this paper at 30°C and 50% relative humidity was observed as early as 2 h after exposure with 50% and 90% mortality observed at 3.5 and 4.6 h (the LT_50_ and LT_90_, respectively) in *M. domestica*. The LT_50_ and LT_90_ increased as the size of the fly species increased (*M. domestica* to *C. macellaria* to *S. bullata*) and increased for all three species when the relative humidity increased from 50 to 70%. These results suggest dehydration was the mode of action. Scanning electron micrographs of *C. macellaria* adults 2 h after diatomaceous earth exposure, showed the flies were evenly self‐covered with the mineral with no obvious damage to the cuticle. Proof of concept was demonstrated that diatomaceous earth as a residual surface treatment could be used as an alternative for filth fly control.

## Graphical abstract

- Mortality for diatomaceous earth was observed as early as 2 h after exposure with 50% and 90% mortality at 3.5 and 4.6 h (LT_50_ and LT_90_, respectively) for *Musca domestica*.
- The LT_50_ and LT_90_ increased when the size of the fly species increased (*M. domestica* to *Cochliomyia macellaria* to *Sarcophaga bullata*) and increased for all three from 50 to 70% relative humidity.
- Results suggest dehydration was the mode of action.

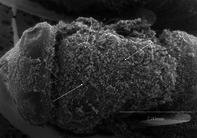

## INTRODUCTION

Flies are nuisance pests of humans and animals. Filth flies breed in animal and human excrement and in almost any kind of decaying organic material including carrion (Graczyk et al., 2001; Greenberg, 1971, 1973; Mingchay et al., 2014; Onwugamba et al., 2018). The fly is contaminated with bacteria, viruses, fungi and parasites from their breeding sites, fly to new locations, and contaminate new sites on contact (Deguenon, Travanty, et al., 2019; Deguenon, Zhu, et al., 2019; Graczyk et al., 2000, 2001, 2005; Greenberg, 1971, 1973; Khamesipour et al., 2018; Mingchay et al., 2014; Onwugamba et al., 2018; Sukontason et al., 2006). The house fly, *Musca domestica* L. (Diptera: Muscidae), is the most well‐known filth fly, physically transporting on the outside of their bodies over a hundred different animal pathogens (Khamesipour et al., 2018), for example, *Escherichia coli* (Szalanski et al., 2004) and *Salmonella* spp. (Pava‐Ripoll et al., 2012). Flies also carry pathogens internally that are released when they regurgitate and defecate (Blazar et al., 2011; Deguenon, Travanty, et al., 2019; Sukontason et al., 2006). In addition to pathogen transmission, two fly species used in this study, the secondary screwworm, *Cochliomyia macellaria* (Fabricius) (Diptera: Calliphoridae) and the grey flesh fly, *Sarcophaga bullata* (Parker) (Diptera: Sarcophagidae), are involved in facultative myiasis (Hall, 1991; James, 1947).

Current methods to control filth flies rely heavily on chemical insecticides with a systemic mode of action, for example, pyrethroids, organophosphates and others (Khan et al., 2013; Mullen & Durden, 2018). Flies are developing resistance to these compounds (Scott et al., 2013). Resource reduction such as cleaning and other cultural practices (e.g., trapping) are useful, but not always sufficient. An alternative approach to systemic chemistry of interest to our group has been applications in the material sciences, including the study of a new mechanical insecticide (MI) made from volcanic rock (VR), Aetna WP (formerly Imergard™ WP). A MI is a mineral that kills by a mechanical mode of action. When the VR was applied as a home, indoor residual wall spray in Africa, VR provided 6 months of control for the mosquito, *Anopheles gambiae* Giles (Diptera: Culicidae) (Deguenon et al., 2020) and was efficacious under practical field conditions against other mosquito species in the United States (Deguenon et al., 2021). VR was effective against sand flies, *Phlebotomus papatasi* (Diptera: Psychodidae) (Chen et al., 2023) and different tick species (Cave et al., 2023; Richardson et al., 2022; Showler et al., 2020; Showler & Harlien, 2023a, 2023b).

Diatomaceous earth (DE, the skeleton of diatoms) is another MI that kills bedbugs (Doggett et al., 2008), beetles (Arthur, 2000; Athanassiou et al., 2004, 2016; le Patourel, 1986; Showler, 2002), fleas (Korunić et al., 2016), cockroaches (Faulde et al., 2006; Hosseini et al., 2014) and thrips (Mitchell et al., 2018). The currently accepted view of the mode of action of DE and MIs in general is abrasion of the outer wax layer and/or absorption of the wax allowing water evaporation causing death by dehydration (Chen et al., 2021; Deguenon et al., 2020; Ebeling, 1971; Lilly et al., 2016; Malia et al., 2016; Mewis & Ulrichs, 2001). However, the rapid death of mosquitoes (Deguenon et al., 2021) and ticks (Richardson et al., 2022) exposed to VR, minimal movement after exposure, and death occurring faster at higher humidity in ticks brings into question this long held view of damage to the wax layer as the method of death. Islam & Rahman (2021) found that DE altered house fly reproductive attributes, but DE has never been studied as a residual surface treatment to kill filth flies. Proof of concept that DE could be used as a residual wall spray like VR to control three common species of filth flies, *M. domestica*, *C. macellaria* and *S. bullata* was investigated. Additionally, the mode of action of DE was considered. One advantage of DE compared to VR for fly control is that DE is already used to control other insects.

## MATERIALS AND METHODS

### Fly rearing

Adults of the house fly, *M. domestica*, the secondary screwworm, *C. macellaria*, and the grey flesh fly, *S. bullata*, were acquired from colonies reared at North Carolina State University (NCSU, Raleigh, NC, USA). *Musca domestica* originally were obtained from Cornell University colonies started in 1982, the *C. macellaria* colony was established at NCSU from field collections in Raleigh, NC, USA, in 2012, and the *S. bullata* colony was started from collections on the NCSU campus, Raleigh, NC, in 2015. All flies were reared with a 16:8 (L:D) cycle at 25 ± 2°C and 50% relative humidity. Adult flies of mixed sex at 8–15 days old after emergence were used for the studies that follow. *Musca domestica* larvae were reared on a homogenous mixture of 1:7.5:15 (v/v/v) of Calf‐Manna (MannaPro®, Manna Pro Products, LLC, St. Louis, MO, USA), wheat germ and distilled water (The Mennel Milling CO., Fostoria, OH, USA) and C. *macellaria* and *S. bullata* larvae were reared on a 1.78:1 (v/v) mix of dry cat food (Purina Cat Chow Complete®, Nestlé Purina PetCare Company, St. Louis, MO, USA) to water (Chen et al., 2021; Deguenon, Zhu, et al., 2019). To facilitate mixing, dry food was added to the water 4 h prior to use.

### Mechanical insecticides and the cone assay

**FIGURE 1:**
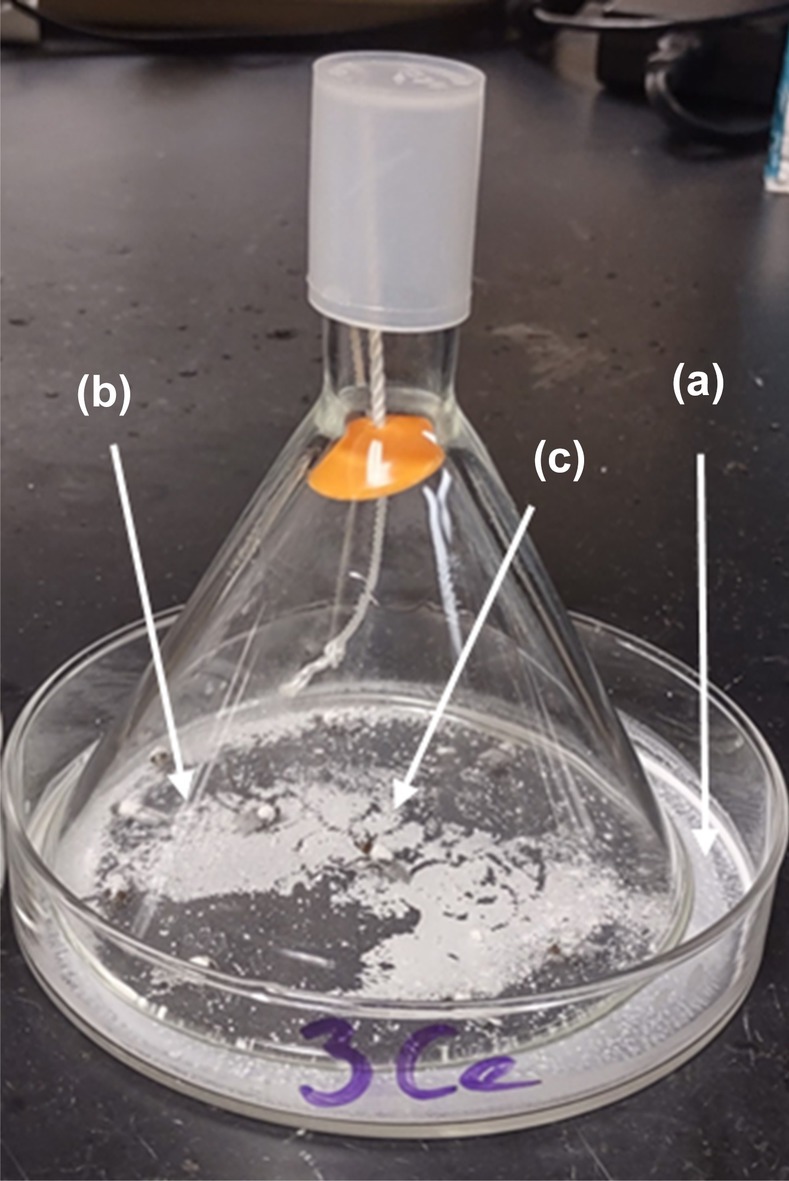
Architecture of the cone test with diatomaceous earth (DE) present on the inside bottom of the Petri dish. (a) the appearance of the coating at the assay start shown outside of the inverted funnel; (b) the appearance of the coating inside of the inverted funnel at the assay completion; and (c), dead *C. macellaria* fly at the assay completion.

DE, Celite 610, was provided by the manufacturer, Imerys (Imerys Filtration Minerals, Inc., Roswell, GA, USA), and was stored in its original packaging at room temperature and humidity until needed. A modified World Health Organization (WHO) cone test (World Health Organization, 2013) was utilized to determine fly mortality (Figure 1). A glass funnel was placed, large opening down, into the bottom of a horizontally positioned glass Petri dish treated with DE (no DE for the control). The funnel had a diameter of 10 cm for the large opening and 1.6 cm for the small opening with a height of 12 cm. Flies inside of the cone (described later) were prevented from flying into the narrow neck of the funnel by an orange stopper held in place with a plastic cap fitted over the outside of the small neck of the funnel (Figure 1). The Petri dish inside bottom was 13.8 cm in diameter and was treated with 5 g of DE/m^2^ (similar to the VR treatment level used as a home, residual wall spray in Africa to control mosquitoes for 6 months; Deguenon et al., 2020). One millilitre of the DE at 74.8 mg/mL in absolute ethanol (Fisher Scientific International, Inc., Hampton, NH, USA) was added to the dish and the dish tilted back and forth to cover the bottom of the dish in toto. The control was treated with 1 mL of ethanol only. All dishes were placed flat on the bench top in a fume hood overnight to allow for complete evaporation of the ethanol. Flies were anesthetized with carbon dioxide gas at room temperature and pressure for 30 s, and 10 flies transferred with soft forceps to each Petri dish bottom after which they were covered by the capped funnel as previously described. Funnels and dishes were placed into a Percival I‐36NL incubator (Percival Scientific, Inc., Perry, Iowa, USA) at either 30°C and 50% relative humidity or 30°C and 70% relative humidity, both with a 16:8 L:D cycle. The number of dead flies was counted every half hour. Flies were counted as dead when they exhibited no movement after light tapping of the funnel with a pencil. Three replicates of three cones per replicate with corresponding controls were performed each day and repeated on two additional days (Cave et al., 2025).

### Scanning electron microscopy (SEM)

Live *C. macellaria* from one cone each (on the same day for the 50% relative humidity condition discussed earlier) for the DE treatment and for the control tests (2 h after the assay started) were anesthetized as described earlier and stored in separate groups (10 flies each) in separate 50‐mL Falcon tubes (Corning Inc., Corning, New York, USA) at −80°C until used. All three species in the cone test quickly become coated in toto as seen by eye without magnification shortly after the start of the assay with no differences found in this coating between species. For this reason and because this was similar to our earlier research with VR against these same flies using the same assay and where the coating was similar between species (Chen et al., 2021), only *C. macellaria* were imaged by SEM in this study. DE SEM was conducted at the Analytical Instrumentation Facility on the North Carolina State University Centennial Campus (Raleigh, NC, USA). The flies were desiccated in a vacuum for 48 h and then mounted with glue onto a Hitachi SEM aluminium mount (Hitachi, Ltd., Chiyoda City, Tokyo, Japan). The samples were covered with a Cressington 108 sputter coater (Cressington Scientific Instruments Ltd., Watford, UK) for 90 s with a 70‐nm gold–palladium mixture (60 Au/40 Pd). A Hitachi SU3900 (Hitachi, Ltd., Chiyoda City, Tokyo, Japan) ultravariable pressure SEM, operated at 50 pascal (50 Pa) was used to image the fly surface.

### *Statistical analysi*s

Time was denoted the independent variable and percentage mortality the dependent variable (± 1 standard error of the mean) used to create line plots comparing treated versus control mortality for each species for each humidity condition. An Abbott's correction (Abbott, 1987) was then applied to each treated data set before the data were used for probit analysis. The Abbott's correction was used to eliminate mortality due to non‐treatment effects. Probit analysis was performed with JMP Pro 17 (JMP Statistical Discovery LLC, Cary, NC, USA). The probit models generated described the probability in hours with a 95% confidence interval when 50% or 90% of each species at each humidity condition would be dead after the start of the treatment (the LT_50_ and LT_90_, respectively). Overlapping confidence intervals between LT_50_s and LT_90_s were considered non‐significant.

## RESULTS

### *Cone assay mortality for* M. domestica

**FIGURE 2:**
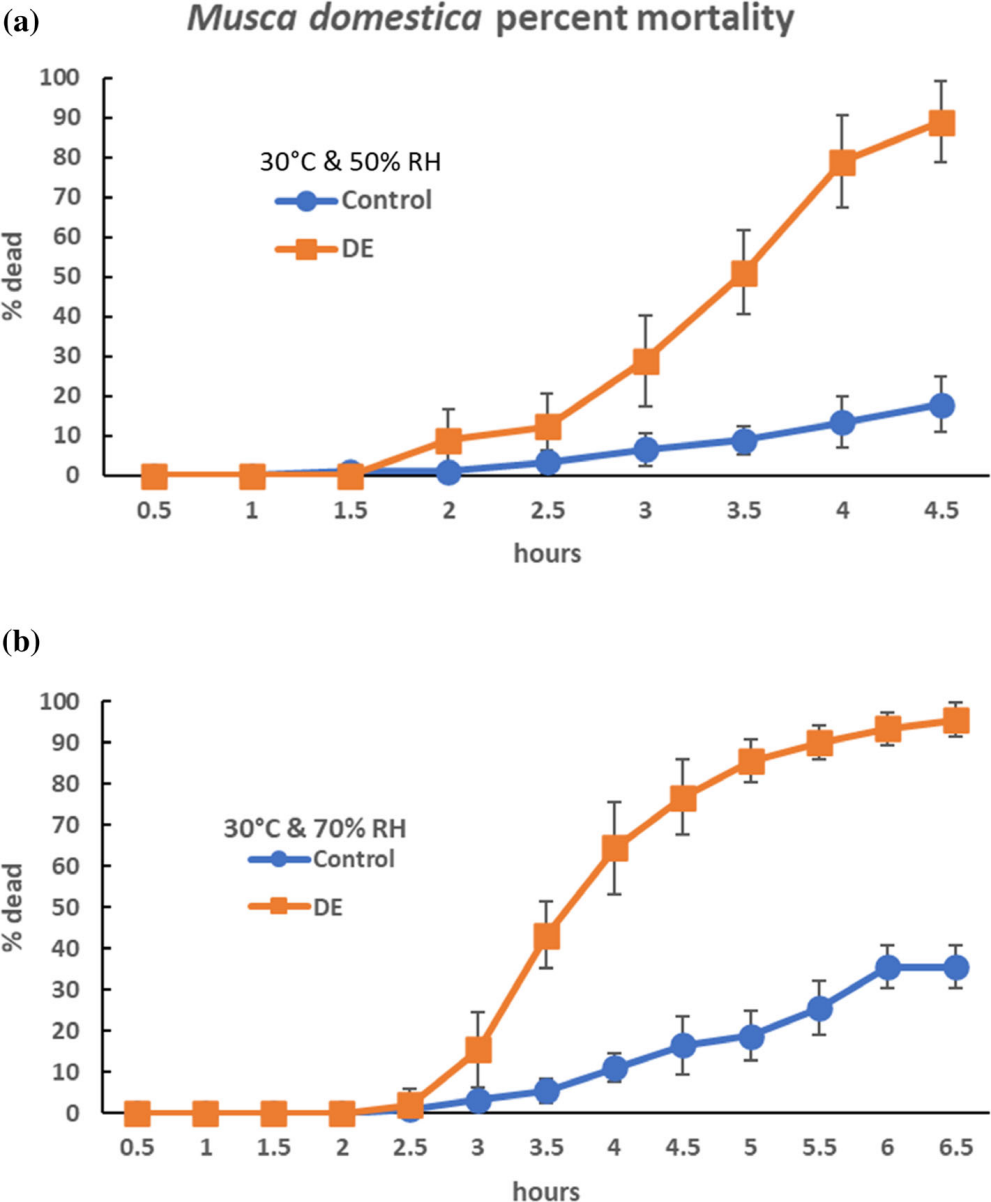
Mortality (not Abbott corrected) versus time of mixed sex, adult house flies, *Musca domestica*, in the cone test (Figure 1) exposed to diatomaceous earth (DE) at (a) 30 ± 1°C and 50 ± 5% relative humidity and (b) 30±1°C and 70 ± 5% relative humidity (incubated during the photophase). Error bars are ±1 standard error of the mean. In some cases, the error bars were too small to be visible.

The first mortality of *M. domestica* at 30±1°C and 50 ± 5% relative humidity for the DE cone test was observed at 2 h with 89% mortality observed at 4.5 h (Figure 2a). Control mortality under these same environmental conditions started at 1.5 h but only reached 18% at 4.5 h. At 30±1°C and 70 ± 5% relative humidity, the first treatment mortality occurred at 2.5 h reaching 96% mortality at 6.5 h (Figure 2b). Control mortality under these same environmental conditions started at 2.5 h and reached 36% at 6.5 h.

Table 1 shows the Abbot corrected mortality and Probit model for the data in Figure 2. The time to 50% mortality (LT_50_) was 3.51 h, and the time to 90% mortality (LT_90_) was 4.61 h for 30±1°C and 50 ± 5% relative humidity. The LT_50_ and LT_90_ for flies at 30±1°C and 70 ± 5% relative humidity were 4.11 and 6.00 h, respectively. The increase in the LT_50_ and LT_90_ at 70% relative humidity was statistically significantly different from these corresponding values at 50% (the 95% confidence intervals did not overlap).

**TABLE 1:** Probit models for time (h) to 50 and 90% mortality (Abbott corrected) for filth flies at 30°C and 50 versus 70% relative humidity (RH) when exposed to DE in a cone test.

Species	RH	*N*	Slope (SEM)	LT_50_ ^a^ (95% CL^b^)	LT_90_ ^a^ (95% CL^b^)	*Χ* ^2^
*M. domestica*	50%	90	1.17 (0.078)	3.51Aa (3.40–3.62)	4.61Ab (4.43–4.84)	85.0
	70%	90	0.86 (0.043)	4.11Ba (3.99–4.23)	6.00Bb (5.42–5.80)	68.9
*C. macellaria*	50%	90	1.12 (0.095)	4.10Ba (3.97–4.26)	5.25Bb (5.00–5.60)	83.2
	70%	90	0.68 (0.032)	4.84Ca (4.70–4.98)	6.73Cb (6.52–6.97)	99.9
*S. bullata*	50%	90	0.46 (0.017)	7.40 Da (7.23–7.56)	10.20Db (9.93–10.5)	36.9
	70%	90	0.23 (0.0064)	11.20Ea (11.0–11.5)	16.70Eb (16.4–17.1)	140.4

^a^ LT_50_ and LT_90_ are the times in h to 50% and 90% mortality, respectively (CL = confidence limit; *Χ* ^2^ = chi‐square).

^b^ Values with overlapping 95% CLs in the same column are not statistically significant and are designated by the same uppercase letter. Lowercase letters inform on statistically significant differences on the same row.

### *Cone assay mortality for* C. macellaria

**FIGURE 3:**
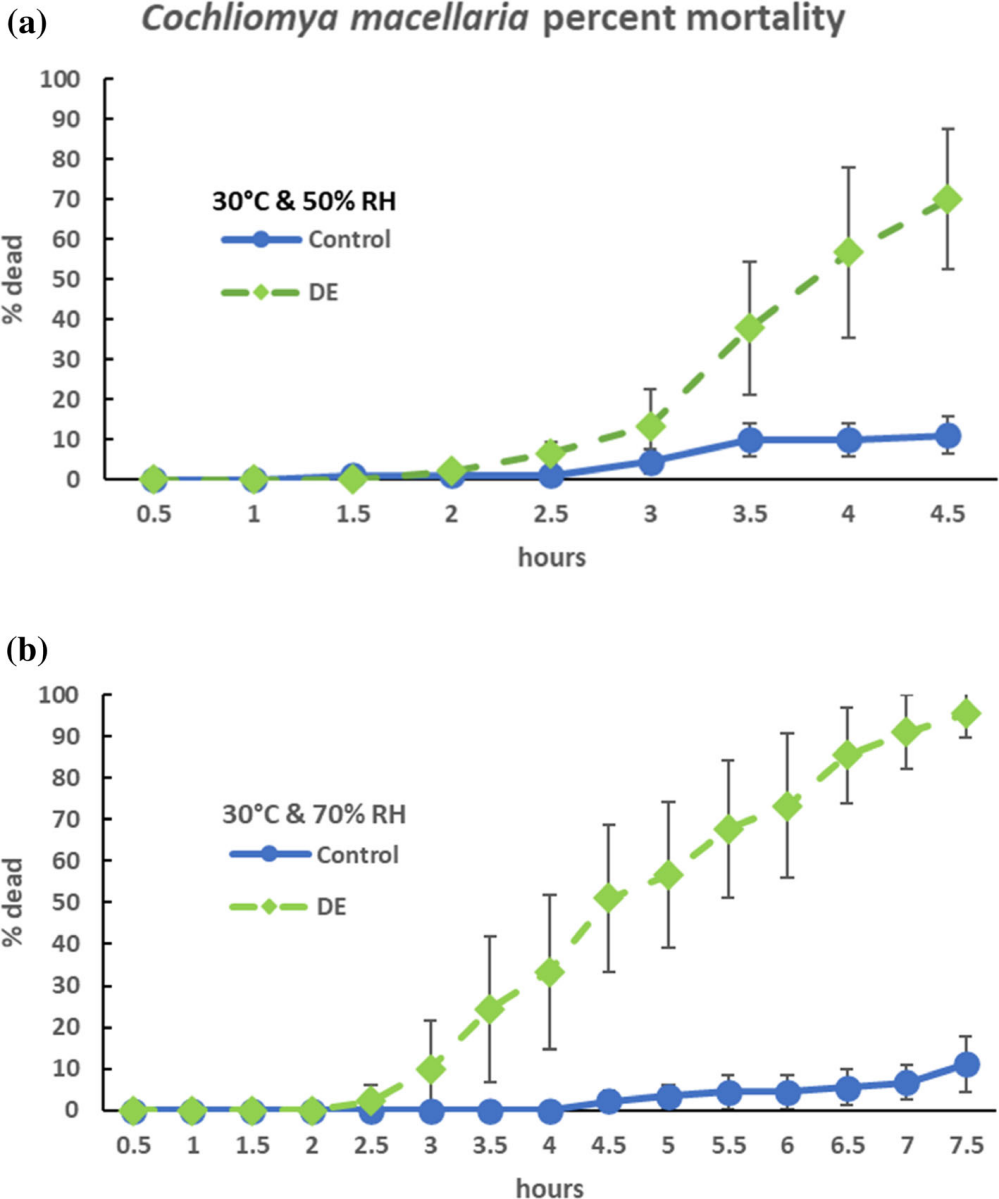
Mortality (not Abbott corrected) versus time of mixed sex, secondary screwworm, *Cochliomyia macellaria*, adult flies in the cone test (Figure 1) exposed to diatomaceous earth (DE) at (a) 30±1°C and 50 ± 5% relative humidity and (b) 30±1°C and 70 ± 5% relative humidity (incubated during the photophase). Error bars are ±1 standard error of the mean. In some cases, the error bars were too small to be visible.

Mortality comparisons for *C. macellaria* adults exposed to DE in the cone test are shown in Figure 3. Flies at 30±1°C and 50 ± 5% relative humidity began dying in the treatment at 2 h with 70% dead at 4.5 h (Figure 3a). Control mortality under these same environmental conditions started at 1.5 h but only reached 11% at 4.5 h. At the same temperature with 70 ± 5% relative humidity, flies started dying in the treatment at 2.5 h and 96% were dead at 7.5 h (Figure 3b). Control mortality under these same environmental conditions started at 4.5 h but only reached 11% at 7.5 h.

The timed mortality data were Abbott corrected and used to develop Probit models (Table 1). The *C. macellaria* LT_50_ was 4.10 h and the LT_90_ was 5.25 h for the 30±1°C and 50 ± 5% relative humidity. At 30±1°C and 70 ± 5% relative humidity, the LT_50_ and LT_90_ were 4.84 h and 6.73 h, respectively. The increase in the LT_50_ and LT_90_ at the higher humidity was statistically significantly different (no overlap of the 95% confidence intervals). Also, at the respective LT_50_s and LT_90_ s and relative humidity, the *C. macellaria* adults survived longer than the house flies.

### *Cone assay mortality for* S. bullata

**FIGURE 4:**
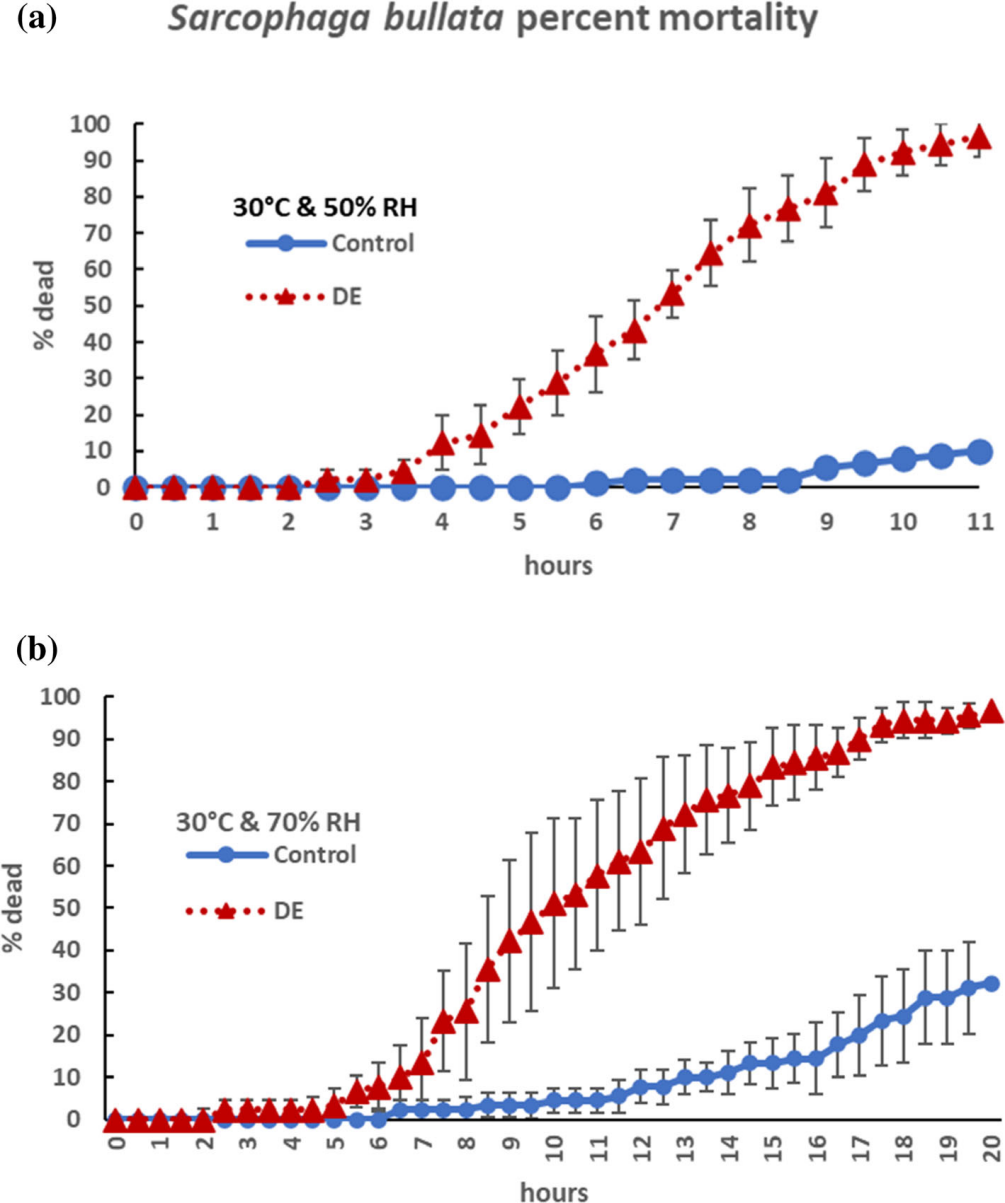
Mortality (not Abbott corrected) versus time of mixed sex, grey flesh fly adults, *Sarcophaga bullata*, in the cone test (Figure 1) exposed to diatomaceous earth (DE) at (a) 30±1°C and 50 ± 5% relative humidity and (b) 30±1°C and 70 ± 5% relative humidity. The incubation was started in the photophase, but the last 5 h of observations were made during the scotophase. During the scotophase, the incubator lights were turned on briefly only for the time needed to score mortality. Error bars are ±1 standard error of the mean. In some cases, the error bars were too small to be visible.

For *S. bullata*, mortality at 30±1°C and 50 ± 5% relative humidity began in the treatment at 2.5 h with 97% dead at 11 h (Figure 4a). Control mortality under these same environmental conditions started at 6 h, but only reached 10% at 11 h. At 30±1°C and 70 ± 5% relative humidity, treatment mortality began at 2.5 h with 97% mortality at 20 h (Figure 4b). Control mortality under these same conditions started at 6.5 h and reached 32% at 20 h.

The Probit models with Abbot's correction are shown in Table 1 for *S. bullata*. The LT_50_ and LT_90_ for 30±1°C and 50 ± 5% relative humidity was 7.40 h and 10.2 h, respectively, and the LT_50_ and LT_90_ for 30±1°C and 70 ± 5% relative humidity was 11.2 h and 16.7 h, respectively. The increase in the LT_50_ and LT_90_ at the higher humidity was statistically significantly different (no overlap of the 95% confidence intervals). Additionally, *S. bullata* reached the LT_50_ and LT_90_ later than both *M. domestica* and *C. macellaria* adults at both 50 and 70% relative humidity, respectively.

### *SEM of* C. macellaria

**FIGURE 5:**
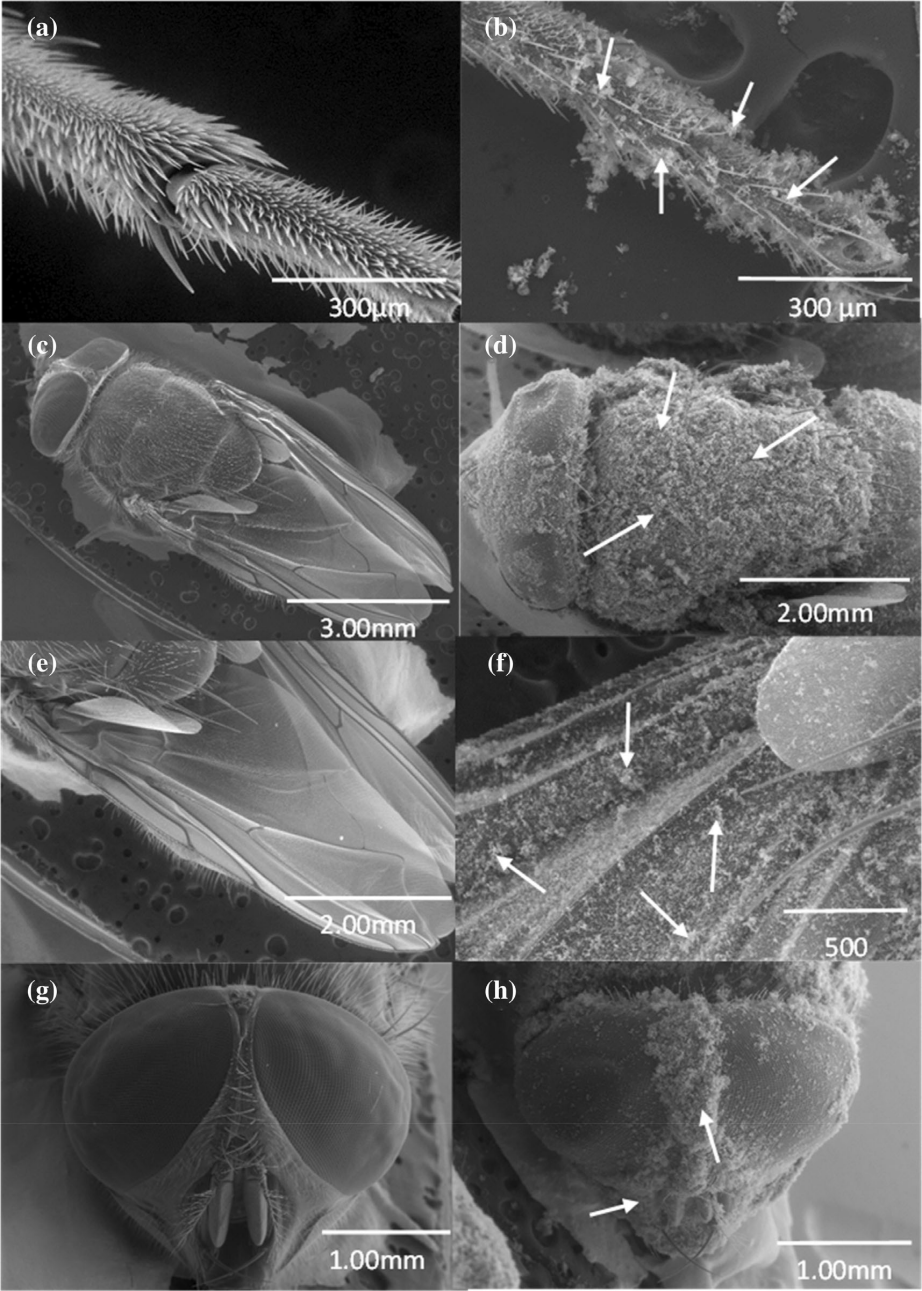
Scanning electron microscopy of adult *Cochliomyia macellaria* from the control cone test (left column) versus those from cones treated with diatomaceous earth (DE) (right column). (a, b) leg, (c, d) body, (e, f) wing and (g, h) head. Arrows are pointing to examples of DE on the insect surface.

**FIGURE 6:**
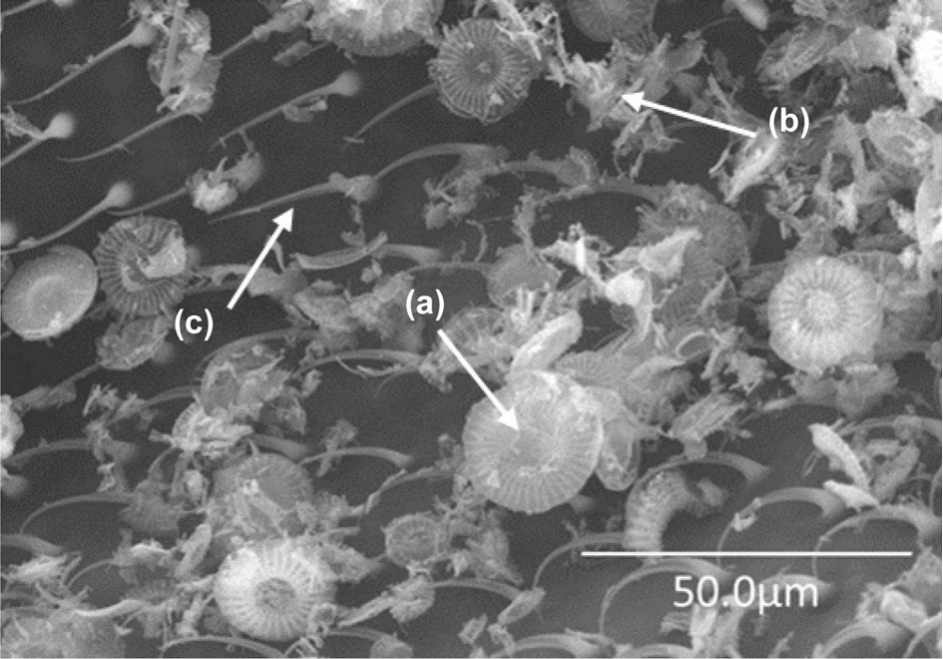
Scanning electron micrograph at a higher magnification showing the morphology of the diatomaceous earth (DE) on the surface of the wing of a secondary screwworm, *Cochliomyia macellaria* fly adult. (a) An intact diatom, (b) a fragment of a diatom and (c) cuticular seta on the surface of the fly wing.

SEM was used to visualize DE on the surface of *C. macellaria*. The control and DE cone test duration was 2 h at 30±1°C and 50 ± 5% relative humidity. This time was selected since this was the beginning when mortality started to appear (Figure 3a). Figure 5 shows DE extensively covering the leg, body, wing and head (Figure 5b,d,f,h, respectively). Figure 6 is a close up of the DE on the wing surface, showing the shape of an intact (Figure 6a) versus fragmented (Figure 6b) diatom skeleton. Note that the setae present on the wing (Figure 6c) did not appear damaged. Also, there was no obvious scarring of the cuticle surface, and the DE appeared to be mostly sitting loosely on the cuticle best seen on the legs (Figure 5a) and wings (Figure 5c) but also on the other parts of the body (Figure 5). These SEM results (Figure 5) were duplicated.

## DISCUSSION

Chen et al. (2021) was the first to examine the potential of using a MI, VR, to control filth flies, with the same cone test used in the current study. The aim of this current paper was to determine if the minerals produced by diatoms, DE, could be used as an alternative to VR for filth fly control. The first appearance of DE mortality for *M. domestica*, *C. macellaria* and *S. bullata* at 30±1°C and 50 ± 5% relative humidity for DE was 2, 2 and 2.5 h, respectively. This was similar to that found for VR using the same bioassay and conditions, that is, 2, 2.5 and 3 h, respectively (Chen et al., 2021). The rapid time to the first appearance of death for both VR and DE was unexpected considering that the hypothesized mode of action for MIs is abrasion of the insect cuticle and/or absorption of cuticle waxes. The time to 50% mortality (the LT_50_) for the DE was 3.51, 4.10 and 7.40 h, respectively (Table 1), which was similar to that found by Chen et al. (2021) for VR with the same fly species, 3.2, 4.3 and 7.1 h, respectively. Deguenon et al. (2021) conducted the same cone test as was used here and by Chen et al. (2021) but with the malaria mosquito, *Anopheles gambiae*, and at a slightly higher temperature (32 ± 1°C) and relative humidity (60 ± 5%) found a LT_50_ of 4.96 h (same time frame as that for filth flies).

As expected, the LT_90_ for DE was longer than that at the LT_50_ for filth flies (Table 1). For example, *S. bullata* at 30±1°C and 50 ± 5% relative humidity had a LT_50_ of 7.40 h compared to the LT_90_ of 10.20 h, a 1.38‐fold increase. The fold increases for the DE were 1.31‐ and 1.28‐fold for *M. domestica* and *C. macellaria*, respectively, under the same conditions and were similar to that of VR as reported before (Chen et al., 2021). These results suggest both DE and VR minerals can provide a high level of control in a reasonable time, even approaching the level of 100% mortality.

In previous research with MIs like DE in other insects (Arthur, 2000; Athanassiou et al., 2004, 2016; Faulde et al., 2006; Hosseini et al., 2014; Korunić et al., 2016; le Patourel, 1986; Mitchell et al., 2018; Showler, 2002), the mechanism of action was hypothesized to be dehydration, suggesting MIs might not be efficacious under high humidity conditions. This was not the case. Increasing the relative humidity to 70% at 30±1°C for *M. domestica* and *C. macellaria* increased the LT_50_ by 0.60 and 0.74 h, respectively, while that for *S. bullata* was longer at 3.8 h (Table 1). Even for the latter, the increase in humidity only increased the time to 50% mortality, 1.5‐fold. This small impact of increased relative humidity for DE was also found for the same species of filth flies (Chen et al., 2021) and for *An. gambiae* (Deguenon et al., 2021) for VR in the cone test. Field studies with VR as a residual, indoor, wall spray in WHO experimental huts in Africa showed 6 months of 80% malaria mosquito control while a deltamethrin positive control was 40 to 45% during the first 4 months decreasing to about 25% in 5–6 months (Deguenon et al., 2020). Similar studies are needed to examine the field use of DE for filth fly control as a residual wall spray. However, the comparisons between DE on filth flies to VR on filth flies and mosquitoes, suggest DE may have a real‐world application for filth fly control that needs further study. The advantage of DE is that an EPA registration is already in place for the use of this MI.

The presumed mode of action of mineral‐based MIs is dehydration that leads to death. The main evidence to support this mechanism of action has been an increase in time to death or decreased mortality when the relative humidity is increased, reported in a number of studies (Chen et al., 2021; Deguenon et al., 2020; Ebeling, 1971; Lilly et al., 2016; Malia et al., 2016; Mewis & Ulrichs, 2001). The exception was the studies of Richardson et al. (2022), where they found the time to death for DE decreased when the relative humidity was increased in nymphal deer ticks, *Ixodes scapularis* Say (Acari: Ixodidae). Garshong et al. (2025) found the same for nymphs of the American dog tick, *Dermacentor variabilis* (Say) (Acari: Ixodidae). In the case of the deer tick, the hypothesis of Richardson et al. (2022) was that the ticks were able to avoid dehydration at the lower relative humidity by reducing their activity (Berger et al., 2014; Sonenshine & Roe, 2014; Vail & Smith, 1998). However, Garshong et al. (Garshong et al., 2025) found DE mostly on the mouth parts of nymphs of the American dog tick, in DE semi field trials, and hypothesized that the increased secretion level of a hygroscopic solution used by the tick to extract water from the air at high humidity was killing the ticks.

Chen et al. (2021) found that the weight of *S. bullata* was 3 and 5 times more than that of *C. macellaria* and *M. domestica*, respectively. The VR LT_50_ and LT_90_ for *S. bullata* in their study were longer than those for *C. macellaria* and *M. domestica*. As the size of an insect increases, the surface area increases by the square and the volume by the cube, reducing the impact of water loss from the surface on dehydration. In the case of *S. bullata*, the larger body mass compared to the surface area would lead to reduced overall body water loss (Kühsel et al., 2017). This trend found for VR with filth fly adults (Chen et al., 2021) also occurred for DE in the current study for the same fly species comparisons, further supporting that the flies were dying from the loss of water from the body surface.

The accepted mechanisms for dehydration after insect exposure to MIs are abrasion of the insect cuticle and/or absorption of lipids from the outer layer of the cuticle (Korunic, 1998; Korunić et al., 2016). The outer layers of the cuticle are covered with wax that prevents water evaporation. Insects are especially vulnerable to water loss because of the large surface area of their internal air‐filled tracheal system that connects to the outside of their bodies for gas exchange by openings in the body cavity called spiracles (Klowden, 2013). In the current study with DE, it was not possible to see the cuticle in many places of the *C. macellaria* adult body because of the high level of mineral coating (Figure 5). The only exception was that of the wing (Figure 5f) where no damage to the cuticle or setae was found near mineral positions. In most cases, it appears the minerals are also loosely covered or suspended above the cuticle by setae (Figure 5), suggesting the level of hard contact between the mineral and cuticle is minimal. This argues against abrasion and wax absorption as mechanisms of action. Deguenon et al. (2021) found that mosquitoes would land on a VR treated surface in a cone test and then shortly afterward become quiescent until they died (Deguenon et al., 2020). Most of the VR by SEM was found on their lower legs. This, along with the lack of mosquito activity and the rapid time to death, was not supportive of abrasion as a mode of action for dehydration. The sides of the thorax were not viewed in the Deguenon et al. (2020, 2021) studies. Ticks (Cave et al., 2023; Richardson et al., 2022), filth flies (Chen et al., 2021), mosquitoes (Deguenon et al., 2020) and sand flies (Chen et al., 2023) were exposed to MI; the resulting SEM photos did not show any cuticular damage. However, due to the right‐angle view to the surface of the cuticle, it is possible that the damage may not be visible as from a cross section of cuticle. However, Garshong et al. (2025) in semi‐field studies with nymphs of the American dog tick, *Dermacentor variabilis* (Ixodida: Ixodidae), found that most of the tick body was devoid of DE, the DE was limited to the mouth parts, and yet some ticks died in minutes.

If abrasion of the cuticle is not a major cause of death, then some other mechanism might be occurring. Richardson et al. (2022) found that nymphal deer ticks, *Ixodes scapularis* (Ixodida: Ixodidae) started dying within minutes after exposure to VR and in a separate experiment to DE. This suggested abrasion was not a mechanism leading to dehydration, since death occurred so fast. Chen et al. (2023) found DE mostly on the sides of the thorax (the location of the spiracles supplying gas exchange to the brain) in sand fly adults, *P. papatasi*, exposed to the mineral in the same cone test used in this current study with filth flies. Richardson et al. (2022) and Cave et al. (2023) found the sieve plate opening into the respiratory tracheal system was sometimes blocked with VR and DE in nymphal and adult *I. scapularis*, and Cave et al. (2023) found the DE inside of the aeropiles of the sieve plate when the ticks were dipped into the dry mineral. Cave et al. (2023) also found that *I. scapularis* adults demonstrated increased locomotor activity after exposure to DE and VR. This conflicting evidence suggests the mode of action for MIs needs re‐examination.

## CONCLUSIONS

Proof of concept was demonstrated to show that the bio‐mineral produced by diatoms was effective in a cone test in killing *M. domestica*, *C. macellaria* and *S. bullata* adults (mixed sexes). Similar studies with mosquitoes using the same cone test were translational for the use of VR (expanded perlite) as a home, indoor residual wall spray for mosquitoes that was effective for 6 months. Time to mortality in the current studies with flies increased with an increase in relative humidity and decreased as the size of the fly species decreased, consistent with dehydration as the mode of action. However, the rapid time to death and apparent minimal hard interactions with the fly cuticle, along with research with mosquitoes, sand flies and ticks where most of the body is devoid of mineral, suggest that the mechanism that MI causes dehydration needs further study.

## AUTHOR CONTRIBUTIONS

**Grayson L. Cave:** Conceptualization; methodology; software; validation; formal analysis; investigation; data curation; writing – original draft; writing – review and editing; visualization. **Kaiying Chen:** Conceptualization; methodology; validation; writing – original draft; writing – review and editing. **Steven S. Denning:** Resources; writing – review and editing. **David W. Watson:** Resources; writing – review and editing. **R. Michael Roe:** Conceptualization; methodology; validation; resources; writing – review and editing; visualization; supervision; project administration; funding acquisition.

## FUNDING INFORMATION

G.L.C. was supported by a research assistantship from the Department of Entomology and Plant Pathology at NC State University as a Ph.D. student. The research was also funded by the Department of the Army, US Army Contracting Command, Aberdeen Proving Ground, Edgewood Contracting Division, Ft Detrick MD, Deployed Warfighter Protection (DWFP) Program Grant W911QY1910003 and by the Research Capacity Fund (HATCH), project award no. 02853, from the U.S. Department of Agriculture's National Institute of Food and Agriculture. Any opinions, findings, conclusions or recommendations expressed in this publication are those of the authors and should not be construed to represent any official USDA or U.S. Government determination or policy.

## CONFLICT OF INTEREST STATEMENT

The authors have no conflicts of interest.

## Data Availability

The data that support the findings of this study are available in the Dryad database, https://doi.org/10.5061/dryad.wpzgmsc31.

## References

[mve70052-bib-0001] Abbott, W.S. (1987) A method of computing the effectiveness of an insecticide. 1925. Journal of the American Mosquito Control Association, 3, 302–303. 3333059

[mve70052-bib-0002] Arthur, F.H. (2000) Toxicity of diatomaceous earth to red flour beetles and confused flour beetles (Coleoptera: Tenebrionidae): effects of temperature and relative humidity. Journal of Economic Entomology, 93, 526–532. 10826209 10.1603/0022-0493-93.2.526

[mve70052-bib-0003] Athanassiou, C.G. , Kavallieratos, N.G. & Andris, N.S. (2004) Insecticidal effect of three diatomaceous earth formulations against adults of *Sitophilus oryzae* (Coleoptera: Curculionidae) and *Tribolium confusum* (Coleoptera: Tenebrionidae) on oat, rye, and triticale. Journal of Economic Entomology, 97, 2160–2167. 15666778 10.1093/jee/97.6.2160

[mve70052-bib-0004] Athanassiou, C.G. , Kavallieratos, N.G. , Chiriloaie, A. , Vassilakos, T.N. , Fătu, V. , Drosu, S. et al. (2016) Insecticidal efficacy of natural diatomaceous earth deposits from Greece and Romania against four stored grain beetles: the effect of temperature and relative humidity. Bulletin of Insectology, 69, 25–34.

[mve70052-bib-0005] Berger, K.A. , Ginsberg, H.S. , Dugas, K.D. , Hamel, L.H. & Mather, T.N. (2014) Adverse moisture events predict seasonal abundance of Lyme disease vector ticks (*Ixodes scapularis*). Parasites & Vectors, 7, 1–8. 24731228 10.1186/1756-3305-7-181 PMC3991885

[mve70052-bib-0051] Blazar, J. , Allard, M. & Lienau, E.K. (2011) Insects as vectors of foodborne pathogenic bacteria. Terrestrial Arthropod Reviews, 4, 5–16.

[mve70052-bib-0006] Cave, G.L. , Chen, K. , Denning, S.S. , Watson, D.W. & Roe, R.M. (2025) GLCave‐WHO cone test mortality data of three filth fly species when exposed to diatomaceous earth. Dryad. Available from: 10.5061/dryad.wpzgmsc31

[mve70052-bib-0007] Cave, G.L. , Richardson, E.A. , Chen, K. , Watson, D.W. & Roe, R.M. (2023) Acaricidal biominerals and mode‐of‐action studies against adult blacklegged ticks, Ixodes scapularis. Microorganisms, 11, 1906. 37630466 10.3390/microorganisms11081906 PMC10457945

[mve70052-bib-0008] Chen, K. , Deguenon, J.M. , Cave, G.L. , Denning, S.S. , Reiskind, M.H. , Watson, D.W. et al. (2021) New thinking for filth fly control: residual, non‐chemical wall spray from volcanic glass. Medical and Veterinary Entomology, 35, 451–461. 33942346 10.1111/mve.12521

[mve70052-bib-0009] Chen, K. , Deguenon, J.M. , Lawrie, R.D. & Roe, R.M. (2023) Biomolecular minerals and volcanic glass bio‐mimics to control adult sand flies, the vector of human *Leishmania* protozoan parasites. Biomolecules, 13, 1235. 37627300 10.3390/biom13081235 PMC10452665

[mve70052-bib-0010] Deguenon, J.M. , Azondekon, R. , Agossa, F.R. , Padonou, G.G. , Anagonou, R. , Ahoga, J. et al. (2020) Imergard™WP: a non‐chemical alternative for an indoor residual spray, effective against pyrethroid‐resistant *Anopheles gambiae* (s.l.) in Africa. Insects, 11, 322. 32456154 10.3390/insects11050322 PMC7290382

[mve70052-bib-0011] Deguenon, J.M. , Riegel, C. , Cloherty‐Duvernay, E.R. , Chen, K. , Stewart, D.A. , Wang, B. et al. (2021) New mosquitocide derived from volcanic rock. Journal of Medical Entomology, 58, 458–464. 32808667 10.1093/jme/tjaa141

[mve70052-bib-0012] Deguenon, J.M. , Travanty, N. , Zhu, J. , Carr, A. , Denning, S. , Reiskind, M.H. et al. (2019) Exogenous and endogenous microbiomes of wild‐caught *Phormia regina* (Diptera: Calliphoridae) flies from a suburban farm by 16S rRNA gene sequencing. Scientific Reports, 9, 1–13. 31889104 10.1038/s41598-019-56733-z PMC6937299

[mve70052-bib-0013] Deguenon, J.M. , Zhu, J. , Denning, S. , Reiskind, M.H. , Watson, D.W. & Roe, R.M. (2019) Control of filth flies, *Cochliomyia macellaria* (Diptera: Calliphoridae), *Musca domestica* (Diptera: Muscidae), and *Sarcophaga bullata* (Diptera: Sarcophagidae), using novel plant‐derived methyl ketones. Journal of Medical Entomology, 56, 1704–1714. 31237324 10.1093/jme/tjz107

[mve70052-bib-0014] Doggett, S.L. , Geary, M.J. & Russell, R.C. (2008) The efficacy of diatomaceous earth against the common bed bug, *Cimex lectularius* . In: Report from the Institute of Clinical Pathology and Medical Research. Australia: Westmead.

[mve70052-bib-0015] Ebeling, W. (1971) Sorptive dusts for pest control. Annual Review of Entomology, 16, 123–158. 10.1146/annurev.en.16.010171.001011 4324088

[mve70052-bib-0016] Faulde, M.K. , Scharninghausen, J.J. & Cavaljuga, S. (2006) Toxic and behavioural effects of different modified diatomaceous earths on the German cockroach, *Blattella germanica* (L.) (Orthoptera: Blattellidae) under simulated field conditions. Journal of Stored Products Research, 42, 253–263.

[mve70052-bib-0017] Garshong, R.A. , Richardson, E.A. , Chen, K. , Cave, G.L. & Roe, R.M. (2025) Use of diatomaceous earth to control nymphal American dog ticks, *Dermacentor variabilis* Say (Acari: Ixodidae): laboratory to simulated field experiments. Experimental and Applied Acarology, 94, 3. 10.1007/s10493-024-00972-x 39638980

[mve70052-bib-0018] Graczyk, T.K. , Fayer, R. , Knight, R. , Mhangami‐Ruwende, B. , Trout, J.M. , da Silva, A.J. et al. (2000) Mechanical transport and transmission of Cryptosporidium parvum oocysts by wild filth flies. American Journal of Tropical Medicine and Hygiene, 63, 178–183. 11388511 10.4269/ajtmh.2000.63.178

[mve70052-bib-0019] Graczyk, T.K. , Knight, R. , Gilman, R.H. & Cranfield, M.R. (2001) The role of non‐biting flies in the epidemiology of human infectious diseases. Microbes and Infection, 3, 231–235. 11358717 10.1016/s1286-4579(01)01371-5

[mve70052-bib-0020] Graczyk, T.K. , Knight, R. & Tamang, L. (2005) Mechanical transmission of human protozoan parasites by insects. Clinical Microbiology Reviews, 18, 128–132. 15653822 10.1128/CMR.18.1.128-132.2005 PMC544177

[mve70052-bib-0021] Greenberg, B. (1971) Flies and disease 1. Princeton, New Jersey: Princeton University Press.

[mve70052-bib-0022] Greenberg, B. (1973) Flies and disease 2. Princeton, New Jersey: Princeton University Press.

[mve70052-bib-0023] Hall, M.J.R. (1991) Screwworm flies as agents of wound myiasis. World Animal Review, 8–17.

[mve70052-bib-0024] Hosseini, S.A. , Bazrafkan, S. , Vatandoost, H. , Abaei, M.R. , Ahmadi, M.S. , Tavassoli, M. et al. (2014) The insecticidal effect of diatomaceous earth against adults and nymphs of *Blattella germanica* . Asian Pacific Journal of Tropical Biomedicine, 4, S228–S232. 25183087 10.12980/APJTB.4.2014C1282 PMC4025297

[mve70052-bib-0052] Islam, M.S. & Rahman, M.M. (2021) Diatomaceous earth‐induced alterations in the reproductive attributes in the housefly Musca domestica L. (Diptera: Muscidae). Elixir Applied Zoology, 96, 41941–41944.

[mve70052-bib-0025] James, M.T. (1947) The flies that cause Myiasis in man. Washington, DC, USA: U.S. Department of Agriculture.

[mve70052-bib-0026] Khamesipour, F. , Lankarani, K.B. , Honarvar, B. & Kwenti, T.E. (2018) A systematic review of human pathogens carried by the house fly (*Musca domestica*). BMC Public Health, 18, 1–15. 10.1186/s12889-018-5934-3 PMC6104014 30134910

[mve70052-bib-0027] Khan, H.A.A. , Shad, S.A. & Akram, W. (2013) Resistance to new chemical insecticides in the house fly, *Musca domestica* L., from dairies in Punjab, Pakistan. Parasitology Research, 112, 2049–2054. 23456023 10.1007/s00436-013-3365-8

[mve70052-bib-0028] Klowden, M.J. (2013) Physiological Systems in Insects, 3rd edition. London, UK: Academic Press.

[mve70052-bib-0029] Korunic, Z. (1998) Diatomaceous earths, a group of natural insecticides. Journal of Stored Products Research, 34, 87–97.

[mve70052-bib-0030] Korunić, Z.K. , Rozman, V. , Liška, A. & Lucić, P. (2016) A review of natural insecticides based on diatomaceous earths. Poljoprivreda, 22, 10–18.

[mve70052-bib-0031] Kühsel, S. , Brückner, A. , Schmelzle, S. , Heethoff, M. & Blüthgen, N. (2017) Surface area–volume ratios in insects. Insect Science, 24, 829–841. 27234132 10.1111/1744-7917.12362

[mve70052-bib-0032] le Patourel, G.N.J. (1986) The effect of grain moisture content on the toxicity of a sorptive silica dust to four species of grain beetle. Journal of Stored Products Research, 22, 63–69.

[mve70052-bib-0033] Lilly, D.G. , Webb, C.E. & Doggett, S.L. (2016) Evidence of tolerance to silica‐based desiccant dusts in a pyrethroid‐resistant strain of *Cimex lectularius* (Hemiptera: Cimicidae). Insects, 7, 74. 27941664 10.3390/insects7040074 PMC5198222

[mve70052-bib-0034] Malia, H.A.E. , Rosi‐Denadai, C.A. , Guedes, N.M.P. , Martins, G.F. & Guedes, R.N.C. (2016) Diatomaceous earth impairment of water balance in the maize weevil, *Sitophilus zeamais* . Journal of Pest Science, 89, 945–954.

[mve70052-bib-0035] Mewis, I. & Ulrichs, C. (2001) Action of amorphous diatomaceous earth against different stages of the stored product pests *Tribolium confusum*, *Tenebrio molitor*, *Sitophilus granarius* and *Plodia interpunctella* . Journal of Stored Products Research, 37, 153–164. 11124378 10.1016/s0022-474x(00)00016-3

[mve70052-bib-0036] Mingchay, P. , Sai‐Ngam, A. , Phumee, A. , Bhakdeenuan, P. , Lorlertthum, K. , Thavara, U. et al. (2014) *Wolbachia* supergroups a and B in natural populations of medically important filth flies (Diptera: Muscidae, Calliphoridae, and Sarcophagidae) in Thailand. The Southeast Asian Journal of Tropical Medicine and Public Health, 45, 309–318. 24968670

[mve70052-bib-0053] Mitchell, R.D. , Mott, D.W. , Dhammi, A. , Reisig, D.D. , Roe, R.M. & Stewart, D.A. (2018) Field evaluation of a new thrips control agent for cotton: a mechanical insecticide. In: Beltwide cotton conference, pp. 786–795.

[mve70052-bib-0037] Mullen, G. & Durden, L. (2018) Medical and veterinary entomology, 3rd edition. Cambridge, MA: Academic Press.

[mve70052-bib-0038] Onwugamba, F.C. , Fitzgerald, J.R. , Rochon, K. , Guardabassi, L. , Alabi, A. , Kühne, S. et al. (2018) The role of ‘filth flies’ in the spread of antimicrobial resistance. Travel Medicine and Infectious Disease, 22, 8–17. 29482014 10.1016/j.tmaid.2018.02.007

[mve70052-bib-0039] Pava‐Ripoll, M. , Pearson, R.E.G. , Miller, A.K. & Ziobro, G.C. (2012) Prevalence and relative risk of *Cronobacter* spp., *Salmonella* spp., and *Listeria monocytogenes* associated with the body surfaces and guts of individual filth flies. Applied and Environmental Microbiology, 78, 7891–7902. 22941079 10.1128/AEM.02195-12 PMC3485945

[mve70052-bib-0040] Richardson, E.A. , Ponnusamy, L. & Roe, R.M. (2022) Mechanical acaricides active against the blacklegged tick, *Ixodes scapularis* . Insects, 13, 672–683. 35893027 10.3390/insects13080672 PMC9331188

[mve70052-bib-0041] Scott, J.G. , Leichter, C.A. , Rinkevich, F.D. , Harris, S.A. , Su, C. , Aberegg, L.C. et al. (2013) Insecticide resistance in house flies from the United States: resistance levels and frequency of pyrethroid resistance alleles. Pesticide Biochemistry and Physiology, 107, 377–384. 24267700 10.1016/j.pestbp.2013.10.006

[mve70052-bib-0042] Showler, A.T. (2002) Effects of kaolin‐based particle film application on boll weevil (Coleoptera: Curculionidae) injury to cotton. Journal of Economic Entomology, 95, 754–762. 12216817 10.1603/0022-0493-95.4.754

[mve70052-bib-0043] Showler, A.T. , Flores, N. , Caesar, R.M. , Mitchel, R.D. & De León, A.A.P. (2020) Lethal effects of a commercial diatomaceous earth dust product on *Amblyomma americanum* (Ixodida: Ixodidae) larvae and nymphs. Journal of Medical Entomology, 57, 1575–1581. 32333017 10.1093/jme/tjaa082

[mve70052-bib-0044] Showler, A.T. & Harlien, J.L. (2023a) Desiccant dusts, with and without bioactive botanicals, lethal to *Rhipicephalus (Boophilus) microplus* Canestrini (Ixodida: Ixodidae) in the laboratory and on cattle. Journal of Medical Entomology, 60, 346–355. 36734019 10.1093/jme/tjad010

[mve70052-bib-0045] Showler, A.T. & Harlien, J.L. (2023b) Lethal effects of Imergard WP, a perlite‐based dust, on *Amblyomma americanum* (Ixodida: Ixodidae) larvae and nymphs. Journal of Medical Entomology, 60, 326–332. 36545899 10.1093/jme/tjac183

[mve70052-bib-0046] Sonenshine, D.E. & Roe, R.M. (2014) Biology of ticks, 2nd edition. New York, NY: Oxford University Press.

[mve70052-bib-0047] Sukontason, K.L. , Bunchu, N. , Methanitikorn, R. , Chaiwong, T. , Kuntalue, B. & Sukontason, K. (2006) Ultrastructure of adhesive device in fly in families Calliphoridae, Muscidae and Sarcophagidae, and their implication as mechanical carriers of pathogens. Parasitology Research, 98, 477–481. 16416126 10.1007/s00436-005-0100-0

[mve70052-bib-0048] Szalanski, A.L. , Owens, C.B. , McKay, T. & Steelman, C.D. (2004) Detection of *campylobacter* and *Escherichia coli* O157:H7 from filth flies by polymerase chain reaction. Medical and Veterinary Entomology, 18, 241–246. 15347391 10.1111/j.0269-283X.2004.00502.x

[mve70052-bib-0049] Vail, S.G. & Smith, G. (1998) Air temperature and relative humidity effects on behavioral activity of blacklegged tick (Acari: Ixodidae) nymphs in New Jersey. Journal of Medical Entomology, 35, 1025–1028. 9835697 10.1093/jmedent/35.6.1025

[mve70052-bib-0050] World Health Organization . (2013) Guidelines for laboratory and field‐testing of long‐lasting insecticidal nets. Document WHO/HTM/NTD/WHOPES/2013.1.

